# Freedom of Speech at St. Bartholomew's Hospital

**Published:** 1913-02-01

**Authors:** G. Acton Davis

**Affiliations:** Acting Treasurer. St. Bartholomew's Hospital, London, E.C.


*To the Editor of* The Hospital.

### Freedom of Speech at St. Bartholomew's Hospital.

*To the Editor of* The Hospital.

Sir,—I should be very greatly obliged if you would be so kind as to give publicity to the accompanying letters in the next issue of your valuable paper.—I am, Sir, yours faithfully,

G. Acton Davis, Acting Treasurer.

St. Bartholomew's Hospital, London, E.C.,
January 23.

